# Primary paranasal sinus hyalinizing clear cell carcinoma: a case report

**DOI:** 10.1186/s13000-017-0659-7

**Published:** 2017-09-25

**Authors:** Batool M. AlAli, Mohammed J. Alyousef, Ahmad Salah Kamel, Mohammad A. Al hamad, Mohammad H. Al-Bar, Roaa M. Algowiez

**Affiliations:** 1 Department of Pathology and Laboratory Medicine, King Fahd Hospital of University, Khobar, Saudi Arabia; 2 College of Medicine, University of Dammam, Dammam, 34212 Saudi Arabia; 3 Department of Otolaryngology, King Fahd Hospital of University, Khobar, Saudi Arabia; 4 Department of Radiology, King Fahd Hospital of University, Khobar, Saudi Arabia

**Keywords:** Hyalinizing clear cell carcinoma, Paranasal sinuses, *EWSR1-ATF1*, Salivary gland, Clear cell carcinoma

## Abstract

**Background:**

Hyalinizing clear cell carcinoma (HCCC) is a rare low-grade tumour of salivary glands that was first described as a distinct entity in 1994 by Milchgrub et al. *EWSR1-ATF1* fusion was found to be specific for this tumour. The majority of the reported cases of HCCC arise from minor salivary glands within the oral cavity. Primary HCCC of the paranasal sinus is extremely uncommon. To our knowledge, only three cases have been reported in the English literature. Herein, we present a case of HCCC of the posterior ethmoid/maxillary sinus.

**Case presentation:**

A 63-year-old lady who presented with a long history of epistaxis. CT scan revealed a destructive mass in the left ethmoid/posterior maxillary sinus extending to the nasal cavity. Surgical excision was done and microscopic evaluation showed a tumour composed mainly of nests of clear epithelial cells separated by fibrocellular and hyalinized septa with extensive bone destruction. The tumour cells expressed CK5/6, EMA and p63 immunohistochemically but were negative for S100 protein, PAX-8, RCC and CK7. Sinonasal renal cell–like adenocarcinomas, myoepithelial carcinoma and metastatic renal cell carcinoma were excluded by radiological and immunohistochemical studies. Fluorescence in situ hybridization analysis revealed an *EWSR1* gene rearrangement. Postoperative radiation was administrated and the patient did not show recurrence or distant metastasis 4 months after the surgery.

**Conclusion:**

Head and neck region have many tumours that demonstrate clear cell changes on histology. Thus, the differential diagnosis for HCCC is wide. Awareness of this rare entity and the possibility of it is arising in unusual location is necessary. *EWSR1-AFT1* fusion, a consistent finding in HCCC, can be used to confirm the diagnosis.

## Background

Hyalinizing clear cell carcinoma (HCCC), which is classified by the World Health Organization (WHO) as “Clear cell carcinoma, NOS” [1], is a rare low-grade salivary gland neoplasm with characteristic clear cells arranged in nests and cords embedded within a hyalinizing stroma. Most of the reported cases of HCCC arise from the minor salivary glands within the oral cavity, with base of the tongue and palate being the most common sites [2, 3]. HCCC of paranasal sinuses is an extremely rare tumour, and up to the best of our knowledge, only three cases have been reported in the English literature [4, 5] (Table 1). Herein, we report a case of HCCC arising primarily in paranasal sinuses in a 63-year-old woman. The diagnosis was molecularly confirmed with *EWSR1* gene rearrangement.

**Table 1:** Reported cases of paranasal sinus HCCC

Author	Location	Age (Years)	Gender	EWSR/Rearrangement	Treatment	Follow up
Davina Stasia Teo et al. (2015) [4]	Right ethmoid sinus extending into the nasal cavity.	69	Male	Unknown	Surgical excision	No recurrence after 10 months
Jui Lan et al. (2017) [5]	Right maxillary sinus.	48	Male	Positive	Surgical excision	No recurrence after 9 months
Jui Lan et al. (2017) [5]	Right maxillary sinus.	80	Male	Positive	Surgical excision + radiotherapy	Not provided
Present case	Left ethmoid/posterior maxillary sinuses.	63	Female	Positive	Surgical excision + radiotherapy	No recurrence after 4 months

## Case presentation

A 63-year-old lady was referred from ER to the ENT department, complaining of recurrent epistaxis episodes for the past 4 years. She also has history of left sided nasal obstruction, facial pressure, yellowish nasal discharge, protrusion of left eye and anosmia. She gave history of weight loss of around 7 kg in the past 3 months associated with decrease of appetite.

Bilateral nasal endoscopic examination showed a mass that easily bleeds on touch, filling the left nasal cavity and pushing the nasal septum to the other side.

**Fig. 1:**
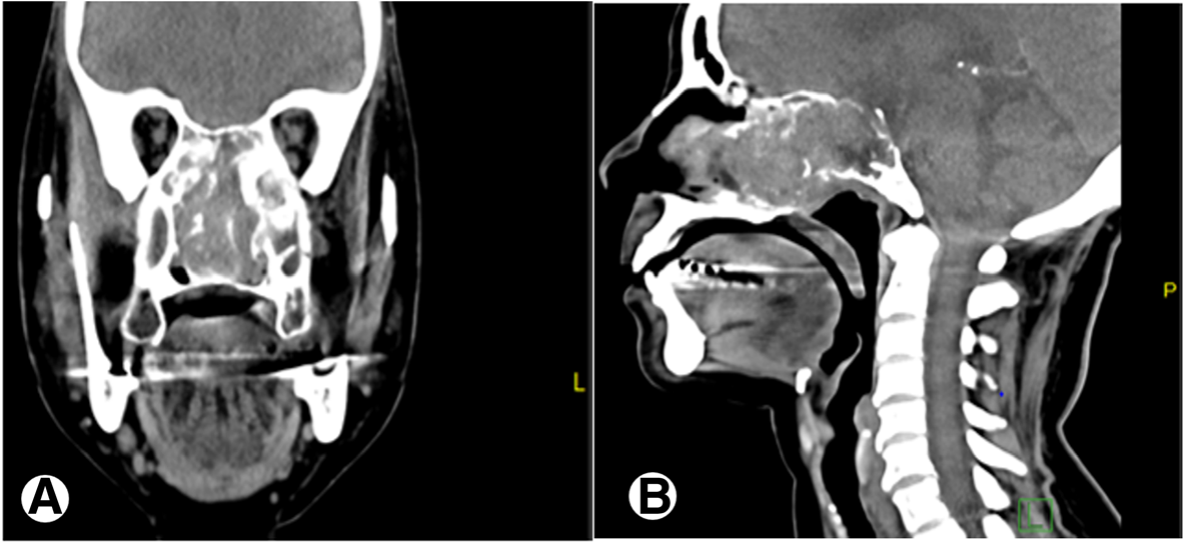
Head CT scan. Coronal head CT scan showing a destructive lesion within the paranasal sinuses extending to the nasal cavity. The epicenter of the mass is seen within the left ethmoid/posterior maxillary and sphenoid sinus (**a**) with extension posteriorly to involve the anterior superior border of the clivus (**b**)

Computed tomography scan (CT) revealed a destructive mass in the left ethmoid/posterior maxillary and sphenoid sinus extending to the nasal cavity. It measured 4 × 4.5 × 6 cm, with extension posteriorly to involve the anterior superior border of the clivus, invading towards the pterygoid bone and reaching the carotid canal (Fig. 1a and b). MRI showed dural thickening lateral to the optic nerve on the left side but no clear intracranial neither periorbital extension. CT scan of chest, abdomen and pelvis were unremarkable for primary or metastatic tumour.

The tumour was treated with surgical excision; however, complete removal was not possible due to attachment to the optic nerve and carotid sheath. Post-operative radiotherapy was administrated.

Multiple soft/tan and bone fragments were received for histological examination. Frozen section was performed for diagnosis and evaluation of margin status. The frozen section diagnosis was reported as “Malignant epithelial neoplasm with prominent clear cell change”. The tumour was extending to the resection margins.

**Fig. 2:**
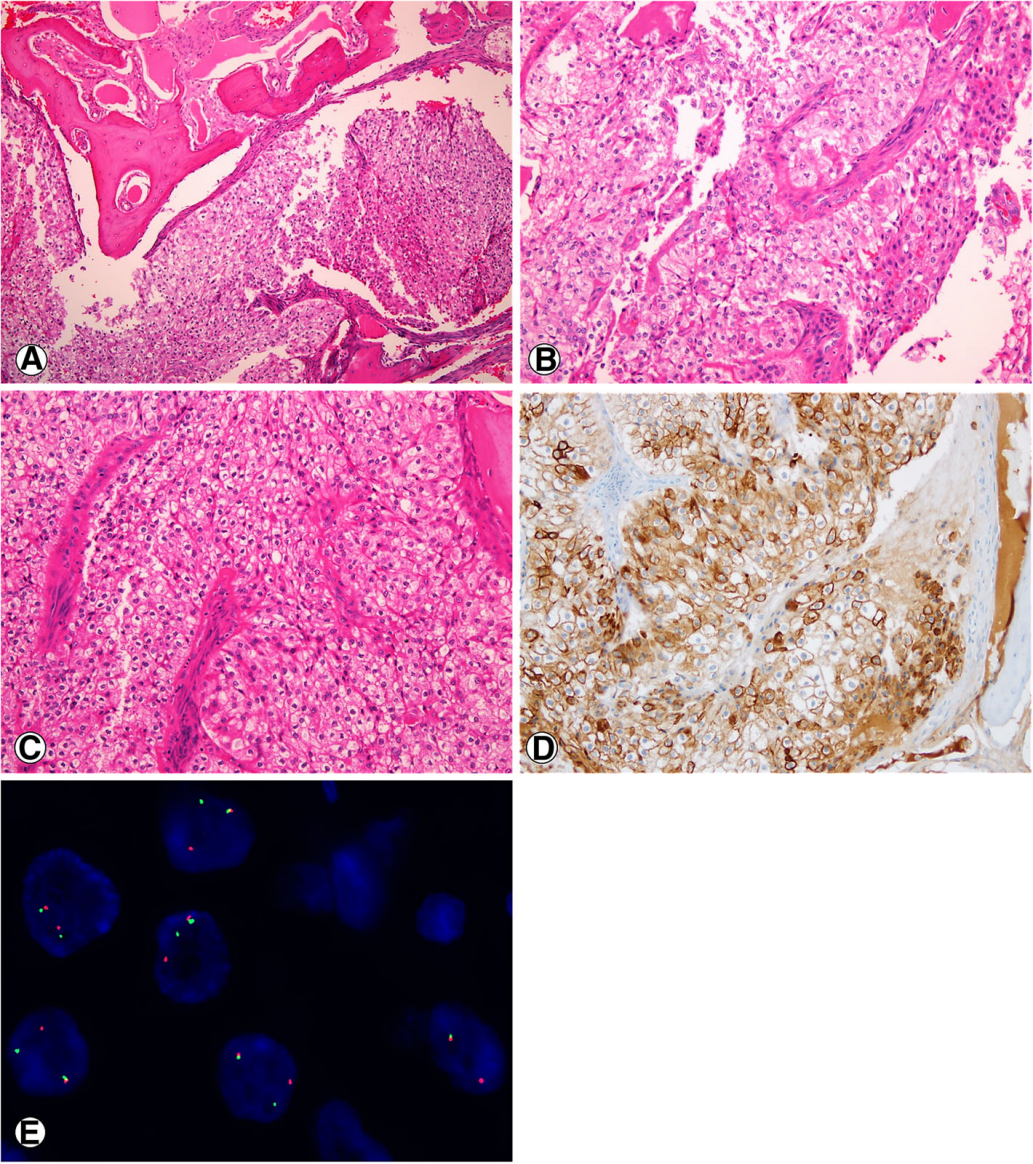
Histological features of HCCC and FISH analysis. **a** - **c**, scanning magnification showing an infiltrative clear cell tumour associated with bone destruction (**a**, H&E, original magnification 40×), these polygonal to round clear cells are arranged in nests separated by hyalinized fibrocellular septa. Other smaller cells with eosinophilic cytoplasm are seen mostly at the periphery of the tumour (**b**, hematoxylin and eosin, original magnification 200×). Nuclei are round and central (**c**, hematoxylin and eosin, original magnification 200×). Diffuse cytoplasmic and membranous staining for CK5/6 (**d**, immunohistochemistry, original magnification 200×). FISH analysis using dual-color, *EWSR1* break apart probe. The tumour cells show one green and one red signal, indicative of a rearrangement of *EWSR1* region (**e**)

Resected tissues were then fixed in 10% buffered formalin and embedded in paraffin. Permanent hematoxylin and eosin (H&E) sections revealed an infiltrative tumour with extensive bone destruction. The tumour consisted of polygonal to round cells arranged in nests and separated by fibrocellular and hyalinized fibrous septa. Most of the cells had a clear cytoplasm while few cells exhibited eosinophilic cytoplasm especially at the periphery of the tumour. The nuclei were round, uniform and centrally located with inconspicuous nucleoli. Mitotic figures and necrosis were not present. Perineural and lymph-vascular invasion were noted (Fig. 2a-c).

The differential diagnosis based on the H&E included squamous cell carcinoma with clear cell changes, mucoepidermoid carcinoma with prominent clear cell change, metastatic renal cell carcinoma and HCCC.

Immunohistochemically, the tumour cells stained positive for EMA, CK5/6 (Fig. 2d), CEA and p63 and were negative for PAX-8, RCC, CK7, SMA and S-100 protein.

FISH analysis of *EWSR1* breakapart probe on paraffin-embedded tumour tissue showed evidence of a 22q12 rearrangement in 164 out of 200 (82%) of interphase nuclei scored (Fig. 2e).

The histological, immunohistochemical and molecular findings were consistent with primary hyalinizing clear cell carcinoma of the paranasal sinuses.

On follow-up, the patient showed no evidence of disease 4 months after the surgery.

## Discussion and conclusions

HCCC is an uncommon salivary gland neoplasm that is slightly predominant in women (female to male ratio = 1.6 to 1) in their seventh (26%) or sixth (20%) decade of life, with the palate and the base of the tongue being the most common sites of the tumour [2]. Occurrence in paranasal sinuses is very rare. Up to our knowledge, only 3 cases in paranasal sinuses have been reported in the English literature.

The clinical presentation varies according to the location of the tumour, and as the most common location of HCCC is the oral cavity, 74.4% of patients presented with a painless submucosal lump in the oral cavity. Mucosal ulcers, pain, dysphagia and nasal obstruction were also reported [2]. In the present case, the patient presented with epistaxis.

HCCC was first described as a distinct entity by Milchgrub et al. [6] in a series of 11 patients with a distinct salivary gland neoplasm that it is characterized histologically by clear cells arranged in cords, trabeculae and nests with distinct cell borders and small bland looking nuclei. The clear cytoplasm was attributed to its glycogen content rather than mucin. Another smaller population of cells with pale eosinophilic cytoplasm and larger nuclei is seen admixed with the clear cell population. In the present case, such cells were mostly clustered at the periphery of the tumour cell nests. Hyalinizing or fibrocellular stroma separating the neoplastic cells is seen in the majority of cases while mitotic figures are usually rare [6, 7]. Lymph-vascular and perineural invasion is seen in 9.2% and 38.3% of the cases, respectively [2]. These findings were present in the current case.

Immunohistochemically, HCCC tumour cells show immunoreactivity for cytokeratins (CK5, CK7, CK8, CK14 and CK19), p63, epithelial membrane antigen (EMA) and carcinoembryonic antigen (CEA) while they are negative for smooth-muscle actin (SMA), muscle-specific actin (MSA), calponin and GFAP. The Ki-67 proliferation index is usually low [2]. PAS stains positive while mucicarmine and PAS-D are negative. This immunohistochemical profile differentiates HCCC from other primary malignancies and metastatic carcinoma.

Sinonasal cancers account for only 3% of all head and neck cancers, with the most common type being squamous cell carcinoma [8, 9]. Sinonasal HCCC has to be differentiated from other more common neoplasms in this region with clear cell changes including: squamous cell carcinoma, sinonasal renal cell–like adenocarcinomas, mucoepidermoid carcinoma, epithelial-myoepithelial carcinoma, myoepithelial carcinoma, acinic cell carcinoma and metastatic carcinoma; particularly, metastatic renal cell carcinoma which is one of the tumours that frequently metastasize to the sinonasal tract [10].

In 2011, Antonescu et al. concluded that *EWSR1-ATF1* fusion is the most reliable tool to differentiate HCCC from other histologically similar tumours [11]. In a review of HCCC by Daniele and his coworkers, *EWSR1-ATF1* fusion gene rearrangment was confirmed in 43 out of 45 cases. This shows the high sensitivity of this test. However, this translocation is not specific for HCCC; as it is also seen in other malignancies including clear cell sarcoma, angiomatoid fibrous histiocytoma and clear cell odontogenic carcinoma (CCOC). The latter is a recently described entity that is identical in histology and immunohistochemical profile to HCCC. In a study of 12 cases of CCOC by Bilodeau et al., they suggest that the location is the most important distinguishing criterion for these tumours, with CCOC regarded as the central or intraosseous counterpart of HCCC [12, 13]. Mucoepidermoid carcinoma, on the other hand, frequently harbor a translocation t(11;19)(q21;p13) resulting in *MECT1-MAML2* fusion. This gene fusion, associated with favourable prognosis, can help in the differential diagnosis [14].

The modality of choice for treatment of HCCC is complete surgical excision with clear margins. The role of post-operative radiotherapy is still not yet proven [2]. Assessment of regional lymph nodes for metastasis is recommended [15].

Even though that this tumour is designated as low-grade, many cases were reported with lymph node, lung and even vertebral metastasis [16–18]. Histological features that may suggest a high-grade tumour include: atypical mitosis, necrosis and bizarre neoplastic cells [17, 19]. Patient follow up is recommended even if the tumour is completely excised.

In summary, we report a case of HCCC involving paranasal sinuses primarily and describe its clinical, histopathologic, immunophenotypic, and molecular cytogenetic features. Tumour cell positivity for p63, CK5/6, PAS and negativity for PAX-8, RCC, mucicarmine and PAS-D exclude mucoepidermoid carcinoma, tumours of myoepithelial origin and metastatic renal cell carcinoma.

## Abbreviations

CCOC: Clear cell odontogenic carcinoma
CEA: Carcinoembryonic antigen
CT: Computed tomography
EMA: Epithelial membrane antigen
ENT: Ear, nose and throat clinic
ER: Emergency room
HCCC: Hyalinizing clear cell carcinoma
NOS: Not otherwise specified
SMA: Smooth muscle actin
WHO: World health organization
